# A cross comparison of technologies for the detection of microRNAs in clinical FFPE samples of hepatoblastoma patients

**DOI:** 10.1038/srep10438

**Published:** 2015-06-03

**Authors:** Aniruddha Chatterjee, Anna L Leichter, Vicky Fan, Peter Tsai, Rachel V Purcell, Michael J Sullivan, Michael R Eccles

**Affiliations:** 1 Department of Pathology, Dunedin School of Medicine, University of Otago, 270 Great King Street, Dunedin 9054, New Zealand; 2 Maurice Wilkins Centre for Molecular Biodiscovery, Level 2, 3A Symonds Street, Auckland, New Zealand; 3 Bioinformatics Institute, University of Auckland, New Zealand; 4 Children’s Cancer Research Group, University of Otago, Christchurch, New Zealand; 5 Royal Children’s Hospital, Melbourne, Victoria, Australia

## Abstract

Although formalin fixed paraffin embedded (FFPE) tissue is a major biological source in cancer research, it is challenging to work with due to macromolecular fragmentation and nucleic acid crosslinking. Therefore, it is important to characterise the quality of data that can be obtained from FFPE samples. We have compared three independent platforms (next generation sequencing, microarray and NanoString) for profiling microRNAs (miRNAs) using clinical FFPE samples from hepatoblastoma (HB) patients. The number of detected miRNAs ranged from 228 to 345 (median=294) using the next generation sequencing platform, whereas 79 to 125 (median=112) miRNAs were identified using microarrays in three HB samples, including technical replicates. NanoString identified 299 to 372 miRNAs in two samples. Between the platforms, we observed high reproducibility and significant levels of shared detection. However, for commonly detected miRNAs, a strong correlation between platforms was not observed. Analysis of 10 additional HB samples with NanoString identified significantly overlapping miRNA expression profiles, and an alternative pattern was identified in a poorly differentiated HB with an aggressive phenotype. This investigation serves as a roadmap for future studies investigating miRNA expression in clinical FFPE samples, and as a guideline for the selection of an appropriate platform.

MicroRNAs (miRNAs) are a large group of small non-protein coding RNAs which are important regulators of gene expression^1^,^2^. This group of small RNAs are expressed in normal cells at all stages of development, as well as in cancer cells. A number of miRNAs are overexpressed in cancer and have been shown to function as oncogenes. These “oncomiRs” promote cancer development by negatively regulating tumour suppressor genes, as well as genes controlling cell differentiation and apoptosis. Other types of miRNAs are underexpressed in cancer, and frequently function as tumour suppressor genes^3^,^4^. MiRNAs have been suggested to play a role in several cancers including hepatoblastoma (HB)^5^,^6^. More recently miRNA expression signatures have been used to classify cancers and define miRNA markers that predict a favourable prognosis. Establishing libraries of miRNA signatures and expression profiles for different classes of tumours may greatly assist in both the diagnosis and treatment of cancer^7^.

HB is a relatively rare childhood liver cancer (and embryonal tumour) that exhibits characteristic histological features of embryonic development^8^. MiRNA expression profiling of HB has been reported previously in four cases^5^,^9^,^10^,^11^. However, most miRNA studies on HB have been carried out using only a relatively small number of samples, and they have involved investigation of a more focused group of candidate miRNAs, rather than a global approach to profile miRNA expression in HB. The previous studies have identified specific miRNAs as prognostic markers, such as miR-492, which is a potential biomarker in metastatic HB^11^. In addition miR-214, miR-199a, miR-150 and miR-125a were found to be up-regulated in HB compared to normal tissue, while miR-148a was found to be down-regulated in HB compared to normal tissue^5^. Several miRNAs have also been suggested to be independent prognostic markers for HB, and are associated with increased survival; these include up-regulation of miR-21, and down-regulation of miR-222 and miR-224^9^. Studies of a larger number of HB samples might allow stratification of HB according to characteristic patterns of miRNA expression and further investigation might provide useful information on the events leading to tumorigenesis, especially if a large panel of hepatoblastomas from a source such as the SIOPEL study could be investigated.

A major technical challenge in cancer research is to obtain reliable genomic data from archival formalin fixed paraffin embedded (FFPE) tissues. FFPE tissue samples, collected as part of the process of routine clinical medicine, often contain very degraded RNA, and extensively cross-linked nucleic acids and proteins, due to the process of formalin fixation. Formalin treatment preserves structural integrity within cells for staining and assists in the diagnosis of cancer by pathologists, but the associated cross-linking has detrimental effects on nucleic acid integrity, making the isolation of intact RNA exceedingly difficult. Further dysfunction occurs by the addition of paraffin wax to the tissue, which inhibits enzymes such as DNA polymerases. Nevertheless, archival FFPE tissues constitute one of the largest sources of tissue, and its utility would increase many-fold if it could be rendered useful for investigations involving cancer genomics^12^. It is therefore crucial to find robust methods to carry out genomic studies using FFPE samples.

A number of previous studies have investigated miRNAs in tissue samples. Five studies have used a single primary platform, such as microarray, together with validation by qPCR, to compare detection of miRNAs in FFPE and frozen tissues^13^,^14^,^15^,^16^,^17^. An additional two studies have compared multiple different microarray platforms to detect miRNAs in FFPE and frozen tissues^18^,^19^. Two further studies have carried out comparative analysis in non-FFPE tissues across multiple platforms (including digital PCR, qPCR, microarrays and NGS) for miRNA detection^20^,^21^. One study, by Kolbert *et al.*, has compared miRNA profiling in fresh frozen and FFPE tissue^22^. However, Kolbert and colleagues only used one sample, which was obtained from three sources (FFPE, fresh frozen and cell lines) and therefore they were not able to assess the variability and relative abundance of the same miRNAs expressed in a number of different tissue samples.

Although these studies have provided valuable information, multiple characteristics of miRNAs commonly detected in the different platforms have not been adequately addressed. For instance, the extent of variability in miRNA expression profiles across different platforms and between different FFPE samples has not been described previously. Therefore the overall characterisation of miRNA profiling in FFPE tissues is lacking crucial information regarding quantitative and qualitative assessment of miRNA detection, using multiple methods of analysis to identify which platform is best suited for these types of samples. To address this question, we have used three techniques (next generation sequencing (NGS), microarray, and the NanoString nCounter) to detect miRNAs from FFPE tumour samples of three HB patients (Fig. 1). In contrast to the analysis of a single tissue sample, as done by Kolbert and colleagues, we have used three samples initially (with replicates) and then we have extended the analysis to 10 additional samples using the NanoString platform. We provide a detailed analysis of shared detection levels for each sample across the platforms, comparison of theoretical and detected distribution of miRNAs in the genome and analysis of other classes of RNA present in the samples (using NGS). We evaluate detection levels of the miRNAs in each platform, compare technical reproducibility, and determine the shared detection of specific miRNAs between the three platforms.

## Results

### Sample description

Initially three FFPE HB tumour samples (S4, S5 and S6) were chosen for cross platform comparison. The sample choice was based on which samples had the best RIN numbers (Supplementary Figure S2), because we were initially concerned that choosing samples with poor RIN numbers (RIN < 2.0) might affect the quality of the data that we could obtain. Two of the three samples (S5 and S6), displayed a fetal epithelial tumour type and subtype, and S5 was histologically described as “cholangioblastic”. Sample S4 was a combination of epithelial and mesenchymal/fetal. One sample, (S6), contained a mutation in the *CTNNB1* gene (beta-catenin), but mutations were not found in S4 and S5. For the NGS and microarray platforms, these three samples were included with a technical replicate per sample. For the NanoString platform, two out of these three samples (S5 and S6) were analysed. Following this analysis a further 10 HB samples were investigated by NanoString. All 13 HB samples were obtained from Société Internationale d’Oncologie Pédiatrique Epithelial Liver Tumor Study Group (or SIOPEL3) (Supplementary Table S1).

### Quality of the data

The data obtained from the NGS platform for S4, S5 and S6 (carried out in duplicate) were of high quality as indicated by FastQC. The median Phred score of the sequenced bases were >34 through to the 50^th^ sequencing cycle (Supplementary Fig. S1). The GC percentage of the samples ranged from 51 to 61. This result suggests that although FFPE samples might be relatively degraded (as indicated by the RIN value, Supplementary Fig. S2), when compared to RNA samples routinely used in other applications, it is possible to obtain high quality sequence reads from FFPE material for miRNA profiling. For the microarray data, several aspects of data quality were inspected, including distribution of fluorescent intensities across the arrays and comparison of differences in individual probe intensities in each array. In the reconstructed pseudo-coloured images of the arrays, the distribution of the fluorescence intensities of the arrays and plots of the spike-ins indicated that good quality data were obtained from these samples (Supplementary Fig. S3 and Fig. S4). For the NanoString platform, the quality of the data was assessed based on the four default parameters (imaging, binding density, positive control linearity and positive control limit of detection). S5 passed all four parameters, but S6 did not pass the positive control limit of detection.

### Comparison of miRNA detection level between platforms

From the MiSeq NGS platform, a total of 6.7 million reads (median=1.2 million) were obtained from the three samples (including technical replicates). After removal of the adaptor sequences and bad quality reads, a total of 4.6 million analysable reads were obtained. Unique alignment efficiency percentages ranged from 29% to 60% (median=55.5%) (Supplementary Table S2) of reference mature miRNAs.

**Figure 2:**
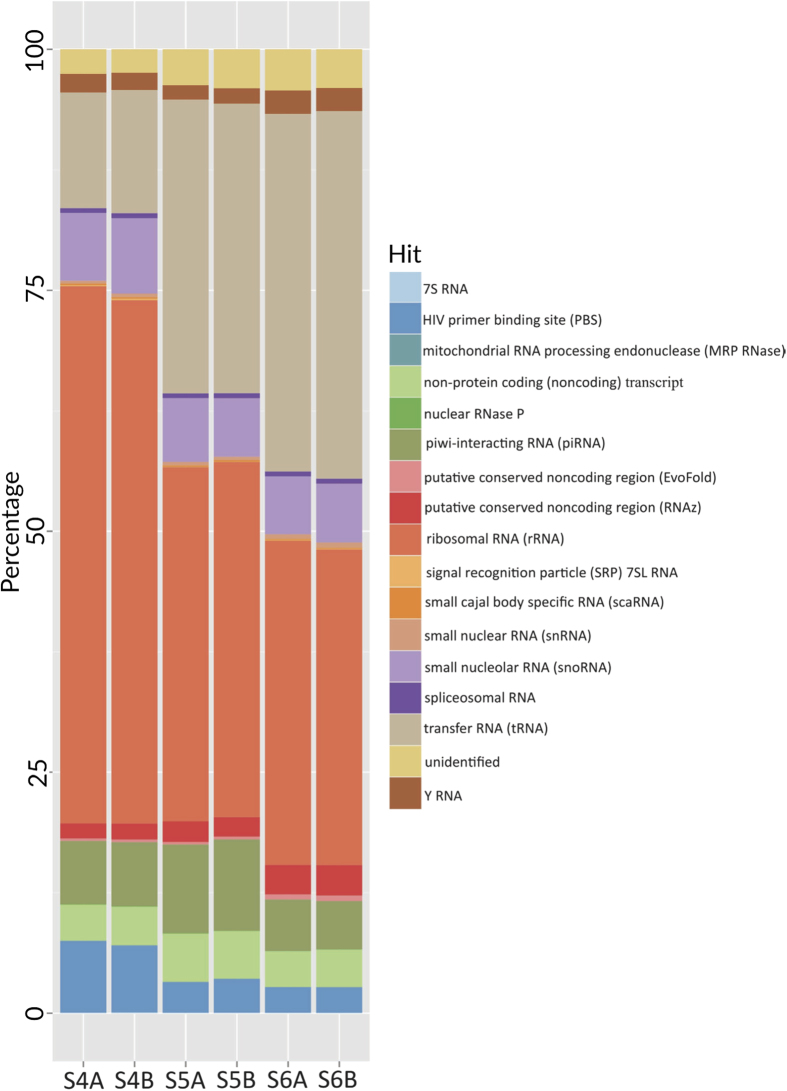
The proportion of different types of RNA (except miRNA) detected within each sample by NGS platform. Sequences were BLAST searched against NCBI Human RefSeq RNA database and the top hit of each query sequence was retrieved and summarised based on the type of RNA.

The length distribution of uniquely aligned reads revealed that the majority of reads were of of 19–22 nucleotides in length, which fits with the length of known miRNAs^23^ (Supplementary Fig. S5). However, the uniquely mapped sequences also showed a second peak at ~30 bp, suggesting the presence of other RNA species apart from miRNAs. To determine the source of these 30 bp sequenced reads, we conducted a BLAST analysis of these sequences and aligned them against other known human RNAs. Many of these sequences lacked annotation in the Genbank small RNA database. Therefore, the small RNAs were annotated using a functional RNA database (http://www.ncrna.org/frnadb/). The majority of these sequences aligned to different classes of ribosomal RNAs (particularly to 5S, 18S, 28S and 45S subunits) and to transfer RNAs (tRNA) (Fig. 2). In addition, several other classes of RNAs were identified (such as piwi-interacting RNA, Y RNA, small nucleolar RNA).

From the uniquely aligned sequence reads, mapped read counts for each miRNA were then assigned. Using an arbitrary threshold of ≥5 mapable reads for each miRNA, a range of 274–408 miRNAs for the three samples, including technical replicates (median=351) was identified. With an increased threshold of ≥10 mappable reads, 228–345 miRNAs were identified in these samples (median=294) (Table 1). The number of detected miRNAs per sample was positively correlated with the number of aligned reads (Pearson’s *r*=0.71), suggesting that sequencing at higher depth would be likely to increase the detection of other miRNAs in these samples (Supplementary Fig. S6). A total of 207 miRNAs were detected in all three samples (and technical replicates) using the NGS platform (with a read count of ≥10).

From the microarray platform, threshold values of probe intensities were determined using the spike-in control with the smallest concentration, which allowed a list of confidently detected miRNAs for each sample to be produced. The microarray platform was able to detect between 79–125 miRNAs for the three samples plus duplicates (median=112) (Table 2). A total of 72 miRNAs were detected in all three samples (and technical replicates) in the microarray platform.

For the nCounter platform negative controls were used to calculate the threshold for miRNAs detection as previously described (NanoString Technologies I. nCounter Expression Data Analysis Guide). Based on the medium stringency threshold, 364 and 534 miRNAs were detected for samples S5 and S6 respectively. With a high stringency threshold 299 and 372 miRNAs were identified in S5 and S6 respectively (Table 3). A total of 226 miRNAs were detected in common in both samples with high stringency.

The chromosomal distribution of theoretical and detected miRNAs in each platform was compared. Broadly, the detection percentage of miRNAs in each platform roughly followed the theoretical distribution. However, for some chromosomes the detection level in samples deviated markedly from the theoretical distribution (e.g., chromosome 14 and 19) (Supplementary Figs. S7-S9). Mapping the miRNA frequency (from miRBase) to Ensembl revealed that chromosomes 14 and 19 had a higher frequency of miRNAs mapped to those chromosomes compared to the others. It is possible that some families (or clusters) of miRNAs occur in these chromosomes, causing them to be detected more frequently than other miRNAs in these samples.

### Technical reproducibility comparison between platforms

**Figure 3:**
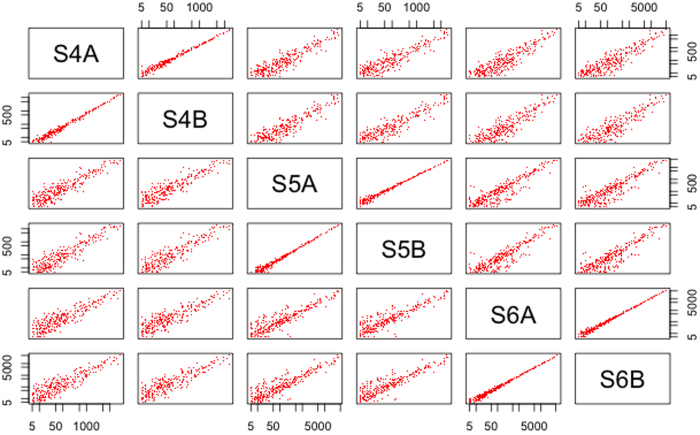
Matrix plot of relationship between different samples in NGS platform. The log of read counts for the detected miRNAs (with a threshold of ≥10 reads for a miRNA) were calculated and plotted on the x and y axis.

**Figure 4:**
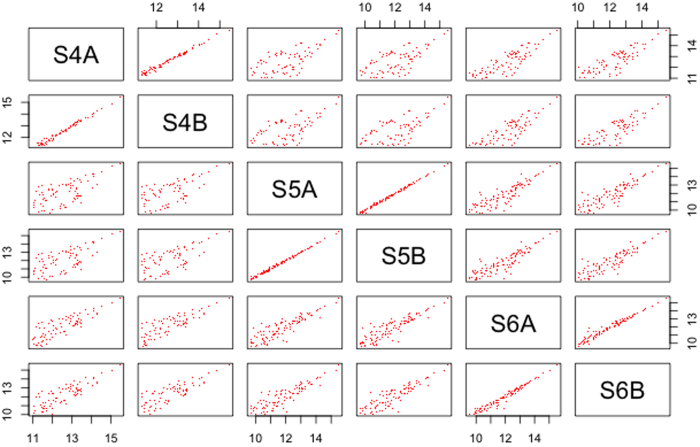
Matrix plot of relationship between different samples in microarray platform. The RMA values for the detected miRNAs were calculated and plotted on the x and y axis.

Next, the reproducibility of the technical replicates was assessed between the platforms. For each of the three samples, technical replicates had been included in the NGS and microarray experiments. For NanoString, technical replicates were not included, as it has been previously shown that nCounter methodology doesn’t require technical replicates, and excellent technical reproducibility using FFPE samples has previously been demonstrated with NanoString^24^. For NGS, all three replicates showed a Pearson’s *r* correlation of >0.99 (for miRNAs with a read count of ≥5). The correlation improved slightly with a higher threshold of read counts (Supplementary Table S3). Similarly for microarray correlations of >0.98 were obtained for all three samples before RMA normalization. The correlation further improved after RMA adjustment for S4 and S5 but not for S6 (Supplementary Table S4). A matrix plot of all the samples included in this study for the NGS and microarray platforms demonstrated that although the technical replicates were highly correlated for miRNA expression level, there was a high level of variability between the samples (Fig. 3 and Fig. 4). Differential expression analysis was beyond the scope of this study, however variability was expected in these samples since they represent different tumour types and *CTNNB1* mutation status and it is likely that other associated molecular changes would be variable among them. Taken together, these results demonstrated that even at lower detection levels, the correlation between replicates was very high, and reproducible results could be obtained from FFPE samples.

### Concordance and shared detection of miRNAs between platforms

**Figure 5:**
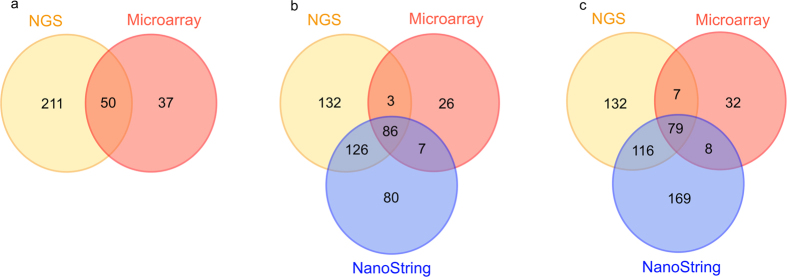
Venn diagrams of shared miRNAs between platforms a) S4 b) S5 c) S6.

The shared detection of miRNAs was investigated between the three different platforms. For this analysis, the list of miRNAs from the replicates (for NGS and array) was combined to produce a single list of detected miRNAs per sample. For samples S4, S5 and S6, 58%, 73% and 68% of the detected miRNAs were common between the NGS and microarray platforms respectively. NGS also shared 71% and 58% of miRNAs for S5 and S6 respectively with NanoString. The shared level of detection was relatively higher between microarray and NanoString (76% and 69% of miRNA for S5 and S6 respectively) (Fig. 5a-c and Supplementary Table S5). The shared detection level between each of these platforms was found to be statistically significant (hyper geometric test, *P* < 0.05, individual *P* values in Supplementary Table S6). One point of note is that NanoString included only 800 miRNAs in the pre-designed chip, whereas the microarray platform had 1735 miRNA probes and NGS obviously could detect any number of miRNAs present in the samples. When the miRNAs detected by microarray and NGS, but not included in the NanoString chip, were removed, there was a higher overlap between the platforms, and in particular, NanoString showed a very high concordance with microarray (Table S7). A total of 98 miRNAs were identified by all three platforms (Supplementary Table S8).

The next question to be investigated was whether the concordance in detection was higher for highly expressed miRNAs, since these miRNAs are likely to be detected more easily. A similar overlap analysis was therefore performed between the top 25% of detected miRNAs for each platform. Surprisingly, the percentage overlap between the platforms decreased when investigating the top 25% of miRNAs detected by each platform (Supplementary Table S5).

**Figure 6:**
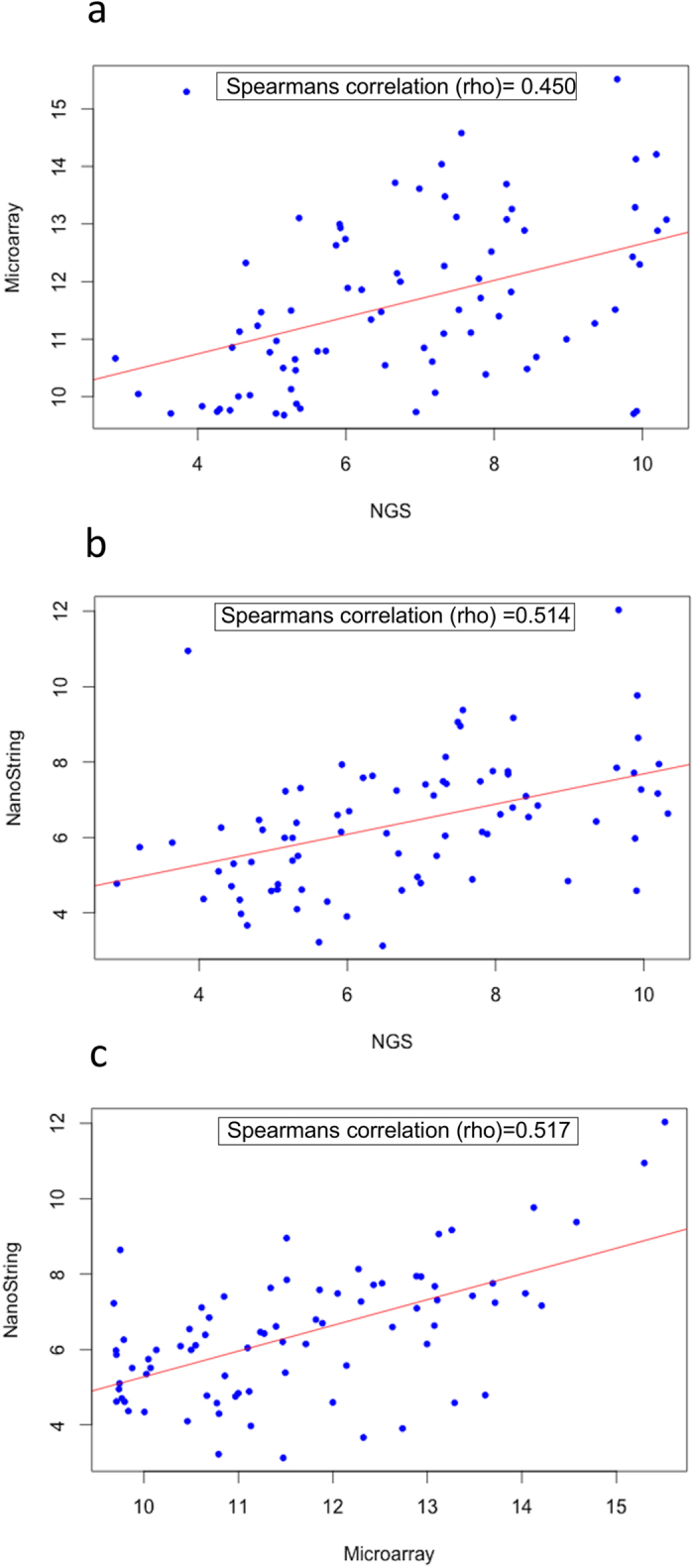
Comparison of the relative abundance levels of the miRNAs detected by all three platforms **(a-c).** Spearman’s ranked correlation (rho) was used to compare relative abundance of the common miRNAs between platforms.

Next, it was examined whether, in the commonly detected miRNAs, the level of expression detected in each platform was similar. For this analysis, the log of read counts (NGS), RMA values (microarray) and nCounter values (NanoString) were plotted against each other to calculate the Spearman rank correlation. Correlation between the results from the microarray and NanoString (Spearman’s *ρ* = 0.517) platforms was stronger than the correlation between NGS and microarray (Spearman’s *ρ* = 0.45) (Fig. 6a-c). The normalization method for the detection of miRNAs differed between each of these techniques, which could account for the differences observed. This was also suggested by the different distributions of the sequenced read counts, RMA and nCounter values for the results of each of the samples analysed (Supplementary Figs. S10-S12).

### Extended analysis with additional samples in NanoString platform

**Figure 7:**
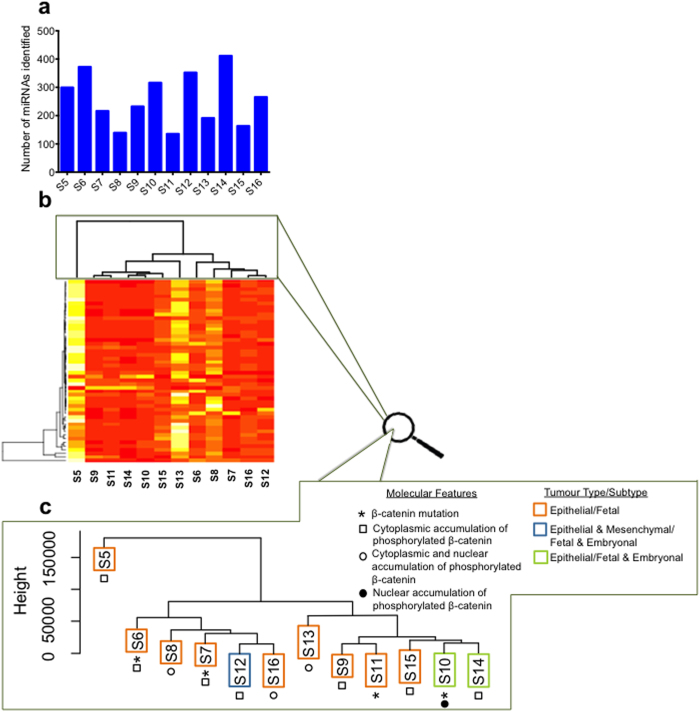
Extended analysis of miRNAs in 10 additional samples with NanoString. **a**) Number of miRNAs detected in each sample with high stringency from the nCounter platform. **b**) Hierarchical clustering of 50 miRNAs detected in all 12 samples using NanoString (method: complete linkage clustering, using a euclidean distance measurement). The heat map shows high (white/yellow) to low (red) expression of the miRNAs in the different samples. **c**) A zoomed in view of the sample clustering together with the clinical phenotype and features associated with each sample.

An additional 10 HB samples were analysed using Nanostring to extend the analysis (sample details in Supplementary Table S1). After filtering, 135 to 411 miRNAs (median=240.5) were detected in the 10 additional HB samples, which is comparable to S5 and S6 (with the exception of S8 and S11) (Fig. 7a). The percentage of shared miRNAs between all 12 HB samples analysed by NanoString was highly significant (*P* < 0.05, hyper geometric test) (individual *P* values in Supplementary Table S9).

Next, hierarchical clustering of 50 miRNAs shared between all 12 samples in NanoString platform was performed (Figs. 7b-7c). Broadly, the HB samples clustered into four clades, with S5 and S13 clustering as two separate clades. S6, S7, S8, S12 and S16 clustered together as a third clade, and S9, S10, S11, S14 and S15 clustered together as a fourth clade. If the same analysis was performed using all 800 miRNAs included in the NanoString analysis (i.e. without filtering miRNAs based on threshold, then again four clades were obtained, and S5 was again the sole member of a separate clade (Supplementary Fig. S14). Interestingly, the patient from which sample S5 was derived had the shortest survival (2 months) of all 13 HB patients, and the HB was noted to have a primitive cholangioblastic histological morphology.

## Discussion and Conclusions

This study has shown that high quality data can be generated, along with high confidence in the detection of miRNAs, even with relatively degraded clinical grade FFPE samples. The three platforms, NGS, microarray and NanoString detected a maximum of 345, 125 and 411 miRNAs respectively. There was some variation in the identities of the miRNAs detected in each platform, with a total of 98 miRNAs concordantly detected by all three platforms. Within each platform reproducibility was very high, and across platforms concordance and shared detection remained high between any two of the three platforms, with significant shared detection occurring between each of the platforms. Since the three platforms involve different methodologies, there is significant potential for methodological bias to influence the results. For example, NGS involves an amplification step, whereas NanoString does not, while microarray involves a hybridization step.

NanoString (nCounter) has previously been used to validate the results of other platforms, including validation of RNA Seq^25^. Thus, the decision of which platform to use to analyse the 10 additional HB samples in this study favoured NanoString. The extended Nanostring analysis then led to the observation of characteristic miRNA expression patterns in HB, and an alternative pattern that was identified in a poorly differentiated HB with an aggressive phenotype. However, the overall preferences as to which platform to use in future studies will be determined by the limitations of each respective platform, particularly with regard to the amount of each sample that is available, the total number of samples to be studied, the research question to be addressed, and the costs associated with using each analytical platform. For example, if the aim is to detect novel miRNAs, then the preferred choice of platform is likely to be NGS. NGS is able to deliver the greatest amount of data and doesn’t require prior knowledge of the sequences to be identified. In addition to miRNAs, NGS can detect other classes of RNAs present in the sample (as shown in Fig. 2). However, NGS requires the largest amount of starting RNA for library preparation, and the results require validation by an additional method. In contrast, NanoString requires smaller amounts of starting material, and can perform miRNA profiling with digital precision, therefore the results do not require further validation by another method. However, it is limited in detection to the screening panel available and may be more expensive when using greater numbers of samples. Microarray falls between the two, but it is also limited to the screening panel available, and it is one of the more expensive platforms (Table 4), although this would depend on sample number.

In conclusion, in this study the strengths and weaknesses of three platforms, for detecting miRNAs, were compared in clinical FFPE samples of HB. An initial analysis of 12 HB samples revealed an alternative miRNA expression pattern in one poorly differentiated HB with an aggressive phenotype. Further studies will be needed to determine the significance of this finding. This investigation serves as a roadmap for future studies investigating miRNA expression in FFPE samples, and as a guideline for the selection of an appropriate platform.

## Methods

### Sample collection

FFPE material from three HB patients was collected for miRNA profiling from the third generation of clinical trials organized by the Société Internationale d’Oncologie Pédiatrique Epithelial Liver Tumor Study Group (SIOPEL3). The age of the patients ranged from 5 months to 1 year 11 months.

### Total RNA extraction and isolation of miRNA from FFPE samples

The FFPE samples were received in a paraffin block and were cut into 1mm cores. Total RNA was extracted using the RecoverALL^™^ Total Nucleic Acid Isolation kit (Ambion, Austin TX, USA) according to the manufacturers instructions. For two of the samples (S5 and S6) total RNA was used for profiling miRNAs in all three platforms. For S4, after total RNA extraction, a miRNA enrichment step was carried out using FlashPAGE Fractionator. This enriched miRNA preparation was then used for profiling S4 in all platforms.

RNA purity was initially assessed on the ND-Nanodrop1000 spectrometer (Thermo Scientific, Wilmington, MA, USA). In addition, the quality and integrity of the RNA were determined using the RNA 6000 Nano chip on the 2100 Bioanalyzer (Agilent Technologies, Palo Alto, CA). The median RNA integrity number for the three samples was 2.2 (Figure S2).

### miRNA library preparation for next generation sequencing and data analysis

For small RNA library preparation 1 μg of RNA was used as input. The libraries were constructed using the TruSeq^®^ Small RNA Sample Preparation kit (Illumina, San Diego, CA) according to the manufacturers guidelines. The technical replicates for the three samples were sequenced in the same flow cell lane. The RNA sequencing was performed on the Illumina MiSeq platform (Illumina) with a single-ended, 50 bp run producing raw FASTQ files.

The quality assessment and processing of sequenced reads were performed as recommended^26^,^27^,^28^. Briefly, the quality of the sequenced reads was assessed using the FastQC application (distributed by Babraham Institute, Cambridge, UK). Adapter sequences from the reads were removed with the Cutadapt software^29^. The processed reads were then mapped to known miRNAs on miRBase 17 with Bowtie1 and miRDeep2 30,31,32. Reference sequences of mature miRNA were used for alignment as it improves the unique alignment efficiency. The number of reads mapped to a miRNA was taken to represent the expression level of that particular miRNA.

### Microarray sample preparation and data analysis

Four hundred nanograms of RNA per sample were labelled without amplification using the Affymetrix FlashTag^™^ Biotin HSR RNA Labeling Kit. The hybridization cocktails were prepared using the Affymetrix Hybridization Wash and Stain Kit and the spike in controls were added from the Eukaryotic Hybridization Control kit. The entire labelled sample was then hybridized on an Affymetrix^®^ GeneChip^®^ miRNA 3.0 array for 16–18 hours at 48 °C in a rotating oven. The microarrays were washed and stained on the fluidics station 450 and scanned in a 3000 7G scanner. The scanned image’s features were converted to numerical values of the probe signal intensity with the Affymetrix GeneChip Command Console software and stored as a CEL file.

CEL files were loaded into the statistical computing environment of R and the Oligo package from bioconductor^33^ was used to perform normalization of the raw probe intensities using a Robust Multi-Array Average (RMA) approach^34^,^35^. The arrays were perfect match only (Pm-only); unmodified Pm intensity values of the probes were used to calculate the median value. As a consequence, the median values of each of the probesets represent the summarized expression values of the transcripts for a particular chip. The threshold for these assays was calculated using the spike-in control with the lowest concentration for each sample. This value is the limit of detection for the microarrays. Any miRNAs below this value were considered not confidently identified (i.e., not beyond noise level) and were removed from further analysis.

Quality of the microarray data was assessed using a miRNAv3 Array QC report conducted in R with bioconducter packages oligo and Affy to create plots for the distribution of probe intensity, spike-in controls, and array comparison. These plots are designed to highlight any batch effects that have influenced the array data^33^,^36^. Boxplots of probe intensities and histograms of log-intensities were generated to compare probe intensity across the arrays. The probe intensity should be extremely similar across all the arrays in these plots. Spike in control plots assess the success of the poly-A tailing and ligation step in the protocol and confirm the lack of RNAses present in the sample. Nine internal “Bio” controls were also added to the array, which provide feedback on the quality of the hybridization, washing and staining procedures performed. The concentration of these nine controls is sequential with BioB being the lowest concentration. It therefore is at the limit of detection for these arrays and it should be present approximately 70% of the time. Lastly, a comparison of the arrays was measured with an MA plot. The MA plots shows the RMA normalised data of all of the possible comparisons between the pairwise log2 intensities of the samples.

### NanoString sample preparation and data analysis

For the NanoString platform, 100 ng of RNA was used as input. Mature miRNAs were ligated to a species-specific tag sequence (miRtag) via a thermally controlled splinted ligation. The unligated miRtags were removed with enzymatic purification, and miRtagged mature miRNAs were then hybridized with an nCounter Human (V2) miRNA Expression Assay CodeSet overnight at 65°C. The unhybridized CodeSet was removed with automated purification performed on an nCounter Prep Station, and the remaining target:probe complexes were transferred and bound to an imaging surface as previously described^37^. Counts of the reporter probes were tabulated for each sample by the nCounter Digital Analyzer, and raw data output was imported into nSolver (http://www.nanostring.com/products/nSolver).

The nCounter^®^ program uses a normalization factor to account for technical noise (such as variations in hybridization, purification, binding efficiency). The normalization factor was generated using the geometric mean of the top 100 miRNAs for each sample; raw counts were multiplied by this sample specific normalization factor to produce the normalized data. In addition, six internal negative controls and six positive controls were included in the nCounter miRNA Expression assays, to correct for background noise. The mean of the negative controls served as the medium stringency threshold for miRNA detection and high stringency was calculated by adding 2SD to the mean of the negative controls (threshold = mean + 2SD).

The quality of the data was assessed using the nSolver program, which uses four parameters (imaging, binding density, positive control linearity, and positive control limit of detection). Imaging refers to the percentage of fields of view (FOV) that have been successfully counted by the digital analyser scan and should remain above 75%. The binding density is the amount of probes that have bound to a portion of the imaging surface. Too many or too few causes inaccuracy in the count numbers, binding densities between 0.05–2.25 spots per square micron was considered good (NanoString Technologies I. nCounter Expression Data Analysis Guide). Positive control probes in the CodeSet were tested for their linearity with a correlation between the concentration of the added target and the resulting count; correlation must be ≥0.95 for high quality data. The limit of detection for each assay was confirmed using the positive and negative controls; the positive control (Pos_E, 0.5fM) was expected to be above the average of the negative control means (NanoString Technologies I. nCounter Expression Data Analysis Guide).

**Other data analysis:** The processed data (read counts for NGS, RMA values for microarray and NanosString values after normalization) were imported in statistical software R (http://www.R-project.org.) to perform statistical analysis and generate related plots.

### Ethics statement

All clinical data and tumour tissue used in this study were collected with informed consent under ethics approvals from the institutional ethics committees of the participating centres and from the human disability and ethics committee (HDEC) of New Zealand (approval numbers are: CTY/01/10/141 and CTY/01/10/142). The experiment was carried out in accordance with approved guidelines.

## Additional Information

**How to cite this article**: Chatterjee, A. *et al.* A cross comparison of technologies for the detection of microRNAs in clinical FFPE samples of hepatoblastoma patients. *Sci. Rep.* **5**, 10438; doi: 10.1038/srep10438 (2015).

## Supplementary Material

Supplementary Information

## Figures and Tables

**Figure 1:**
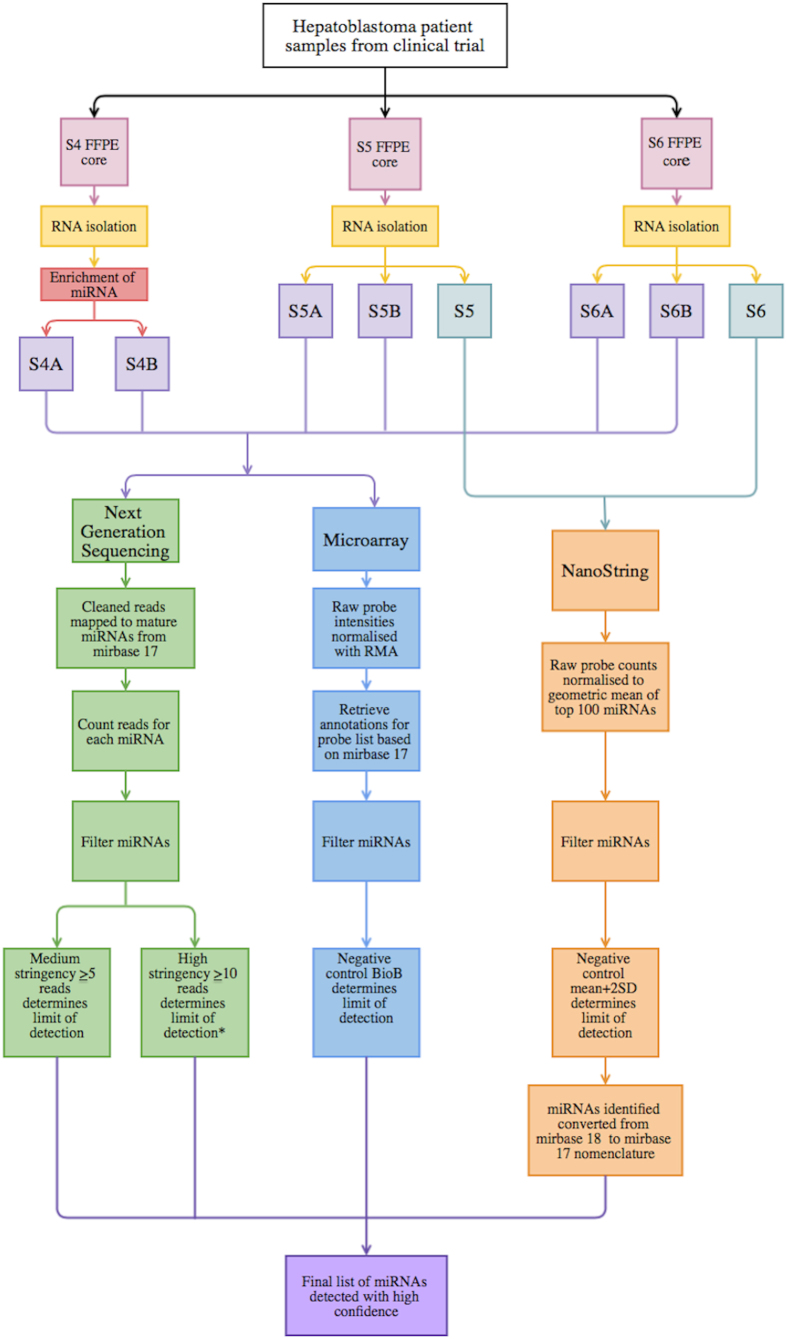
Overall study design and workflow. The * symbol indicates where miRNAs with ≥10 reads were included for further comparative analysis between platforms.

**Table 1:** The number of miRNAs from the NGS platform.

**Sample**	**Number of miRNAs identified with read threshold ≥5**	**Number of miRNAs identified with read threshold ≥10**
S4A	306	260
S4B	274	228
S5A	408	345
S5B	339	274
S6A	385	327
S6B	364	315

**Table 2:** Number of miRNAs detected in the microarray platform.

**Sample**	**Number of miRNAs identified**	**Threshold (RMA adjusted value for BioB-3)**
S4A	87	10.94
S4B	79	11.28
S5A	117	9.68
S5B	119	9.67
S6A	125	9.55
S6B	107	10.06

**Table 3:** Number of detected miRNAs at medium and high stringency from the nCounter platform.

**Sample**	**Number of miRNAs identified (medium stringency)**	**Threshold value**	**Number of miRNAs identified (high stringency**	**Threshold value**
S5	374	16.33	299	21.65
S6	543	16.67	372	23.67

**Table 4:** Cost estimates for the three platforms for miRNA detection experiments.

**Component**	**NGS**	**Microarray**	**NanoString**
RNA input per sample (ng)	1000	400	100
Sample QC	12	12	10
Library construction	305	-	-
Sample prep, hybridization	-	691	393
Sequencing	263	-	-
Total cost per sample^#^	580	703	403

^#^ indicates all costs are shown in US dollars.
