# Erratum: A cross comparison of technologies for the detection of microRNAs in clinical FFPE samples of hepatoblastoma patients

**DOI:** 10.1038/srep13505

**Published:** 2015-09-02

**Authors:** Aniruddha Chatterjee, Anna L. Leichter, Vicky Fan, Peter Tsai, Rachel V. Purcell, Michael J. Sullivan, Michael R Eccles

*Scientific Reports* **5**: Article number: 1043810.1038/srep10438; published online: 07032015; updated: 09022015.

In this Article, the Accession codes were omitted from the Additional Information section:

Data availability: Accession codes: All the data generated and described in this article have been submitted to the NCBI Gene Expression Omnibus (GEO; http://www.ncbi.nlm.nih.gov/geo/). The NGS data were submitted under accession number GSE62010 (URL: http://www.ncbi.nlm.nih.gov/geo/query/acc.cgi?acc=GSE62017), The microarray data were submitted under accession number GSE62011 (URL: http://www.ncbi.nlm.nih.gov/geo/query/acc.cgi?acc=GSE62011) and the NanoString data were submitted under accession number GSE62017 (URL: http://www.ncbi.nlm.nih.gov/geo/query/acc.cgi?acc=GSE62017). These three accession numbers are linked as super series.

