# Supplementary material for: A cross comparison of technologies for the detection of microRNAs in clinical FFPE samples of hepatoblastoma patients
Source: Sci Rep. 2015 Jun 3;5:10438. doi: 10.1038/srep10438 (PMC4453922; doi:10.1038/srep10438)
Supplement: Supplementary Information [file srep10438-s1.pdf]

Supplementary information

(Figures S1- S13, Tables S1- S9)

A cross comparison of technologies for the detection of microRNAs from clinical FFPE samples of hepatoblastoma patients

Aniruddha Chatterjee[1,2#], Anna L Leichter[1#], Vicky Fan[3], Peter Tsai[3], Rachel Purcell[4], Michael J Sullivan[5], Michael R Eccles[1*]

[1]Department of Pathology, Dunedin School of Medicine, University of Otago, 270 Great King Street, Dunedin 9054, New Zealand [2]Gravida: National Centre for Growth and Development, 2-6 Park Ave, Grafton, Auckland 1142, New Zealand [3]Bioinformatics Institute, University of Auckland, New Zealand [4]Children's Cancer Research Group, University of Otago, Christchurch, New Zealand [5] Royal Children's Hospital, Melbourne, Victoria, Australia

# These authors contributed equally to this article.

*To whom correspondence should be addressed. Professor Michael R Eccles, Department of Pathology, Dunedin School of Medicine, University of Otago, P.O. Box 913, Dunedin, New Zealand, Telephone: +64 3 479 7878, E-mail: michael.eccles@otago.ac.nz

Additional Figures

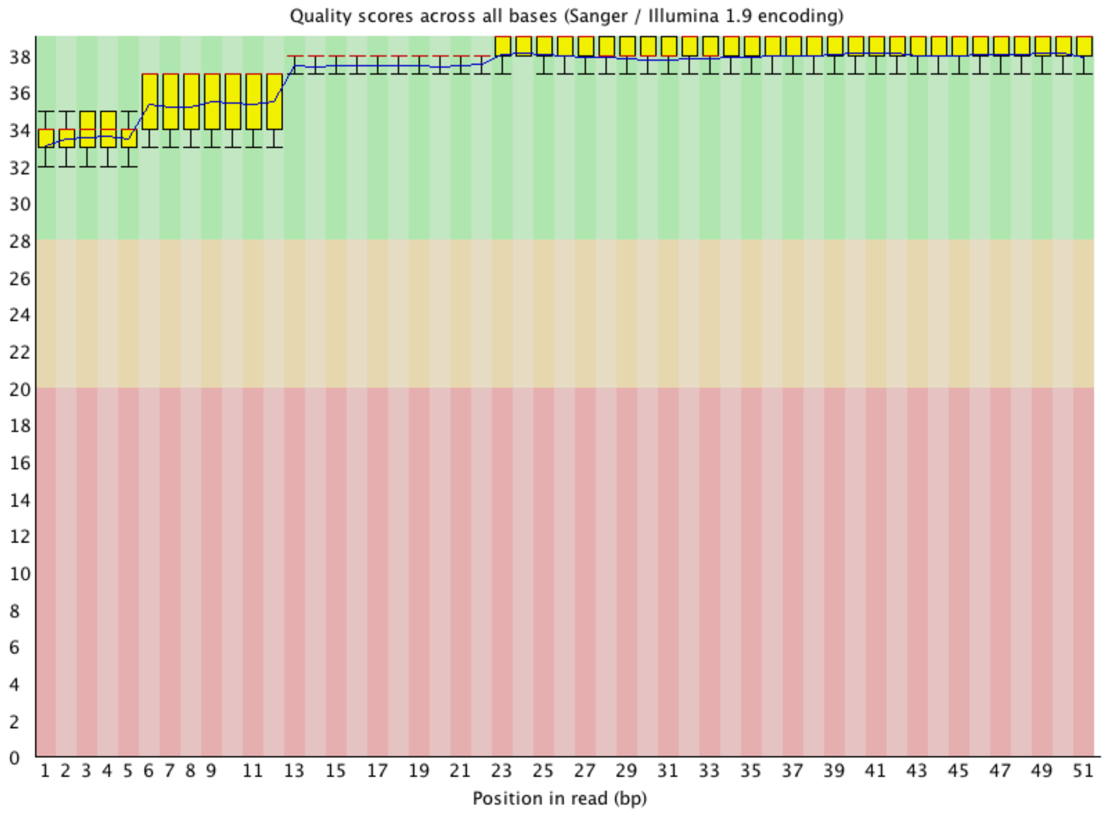

Figure S1: **A representative example of sequence quality of FFPE samples as indicated by FASTQC.** The yellow box plots (red bar: median, box: interquartile ranges 25–75%, and whisker: 10–90% percentile) show the base-calling quality scores across all sequencing reads of sample 2. The blue line indicates the mean quality score. The other samples had similar per base sequence quality.

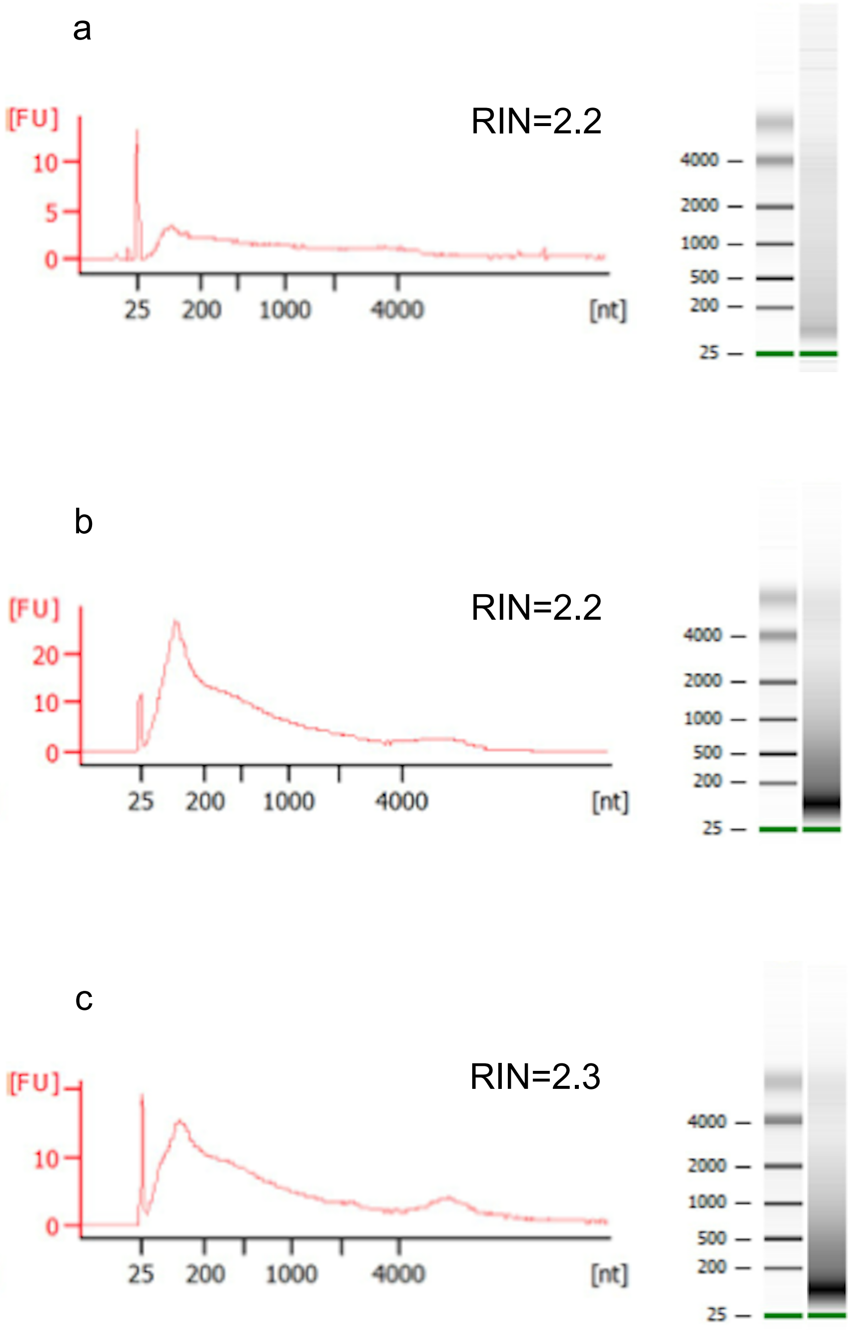

Figure S2: Bioanalyser traces of the 3 FFPE samples used in this study. a) Sample S4 b) Sample S5 c) Sample S6. The y-axis represents florescence units as detected by Bioanalsyer and the x-axis demonstrates the nucleotide length. On the right hand side of the image the corresponding gel electropherogram is shown.

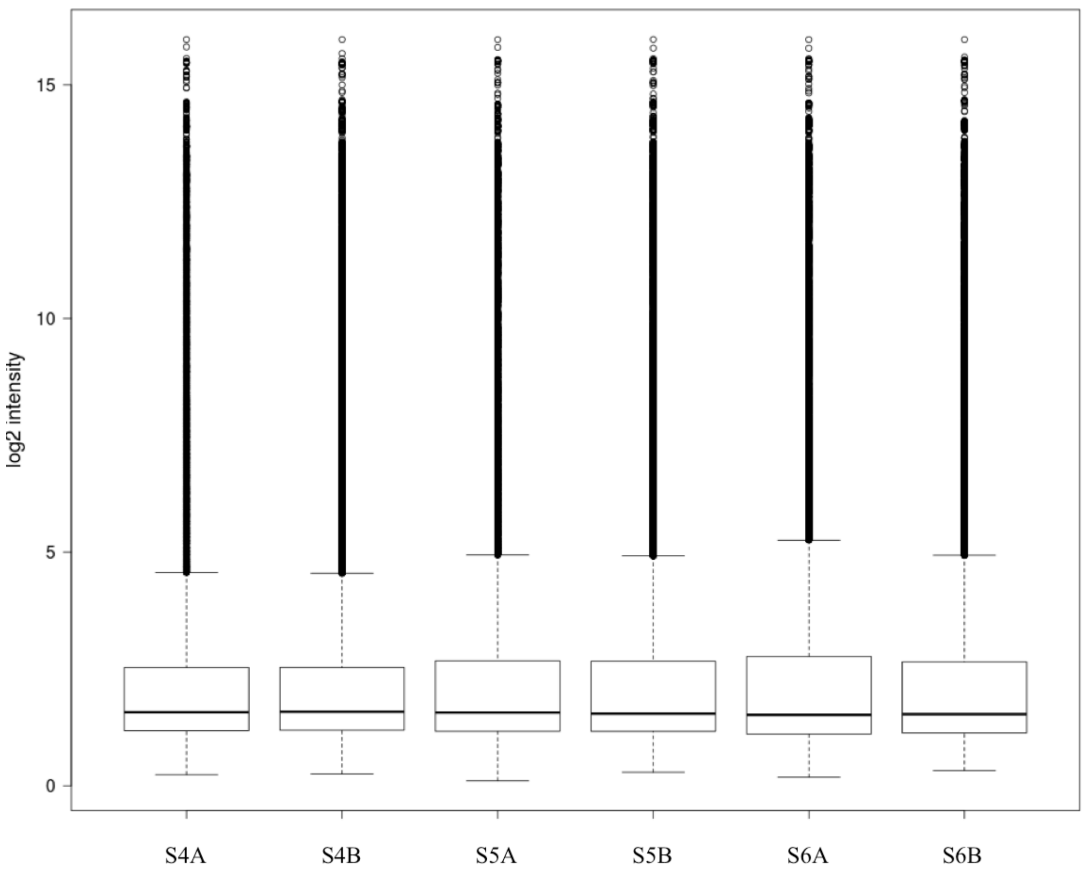

Figure S3: Boxplot of the RMA preprocessed and normalised data. (Suggesting that the distribution of the probeset fluorescence intensities across all of the samples and replicates is quite similar).

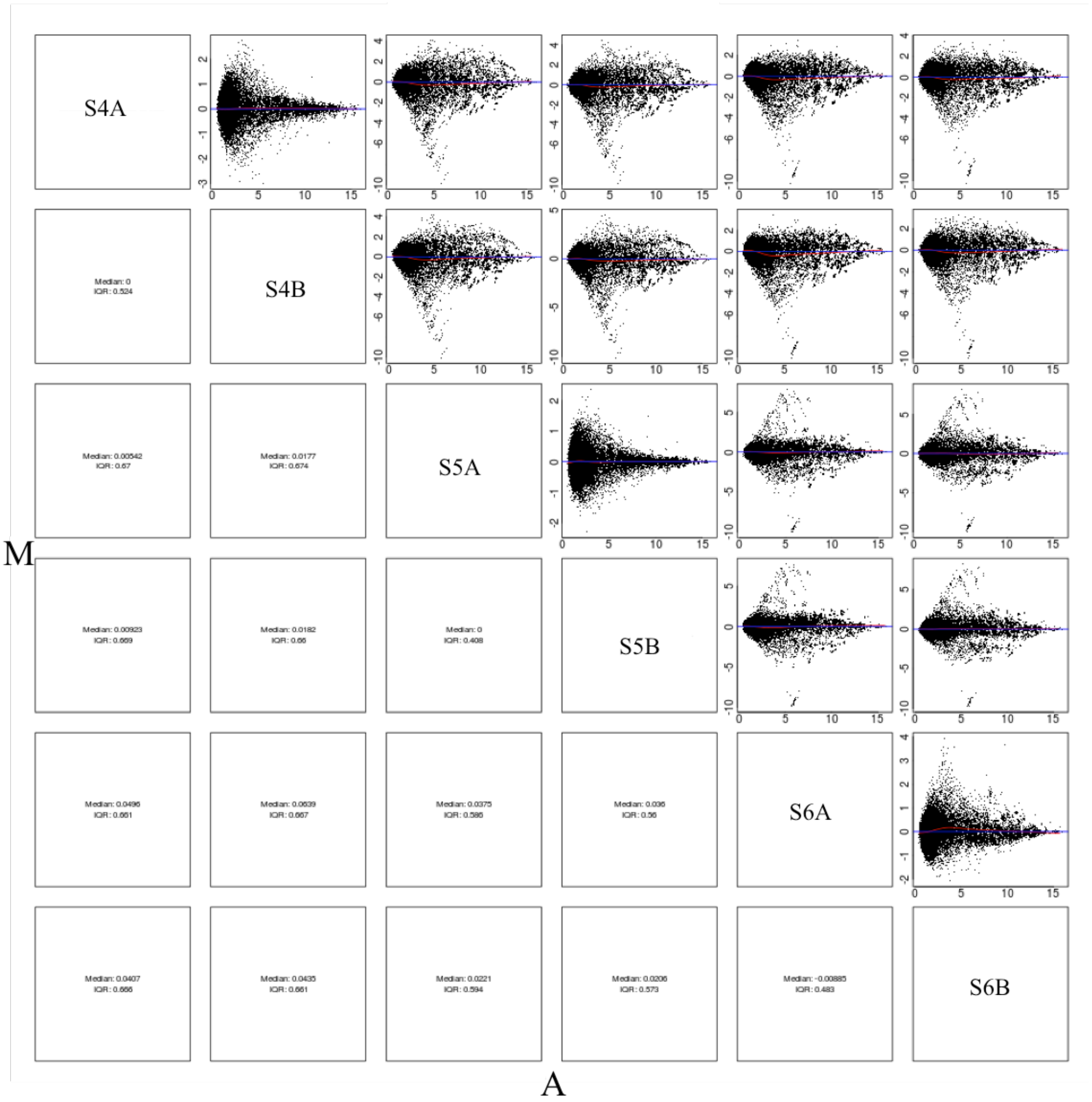

Figure S4: The MA plots of the RMA normalised data show all of the comparisons between the pairwise log2 intensities of all of the arrays. M is the difference between two arrays, and A the average intensity between two arrays. It is assumed that for most probesets present on the microarray, there are no differences in fluorescence intensity between two arrays. We therefore expect to see most of the probes centered on M=0 (blue line) regardless of the average intensity. The red line is the loess line. This plot suggests that the technical replicates are more similar to each other compared to the other samples, but not too dissimilar to each other overall.

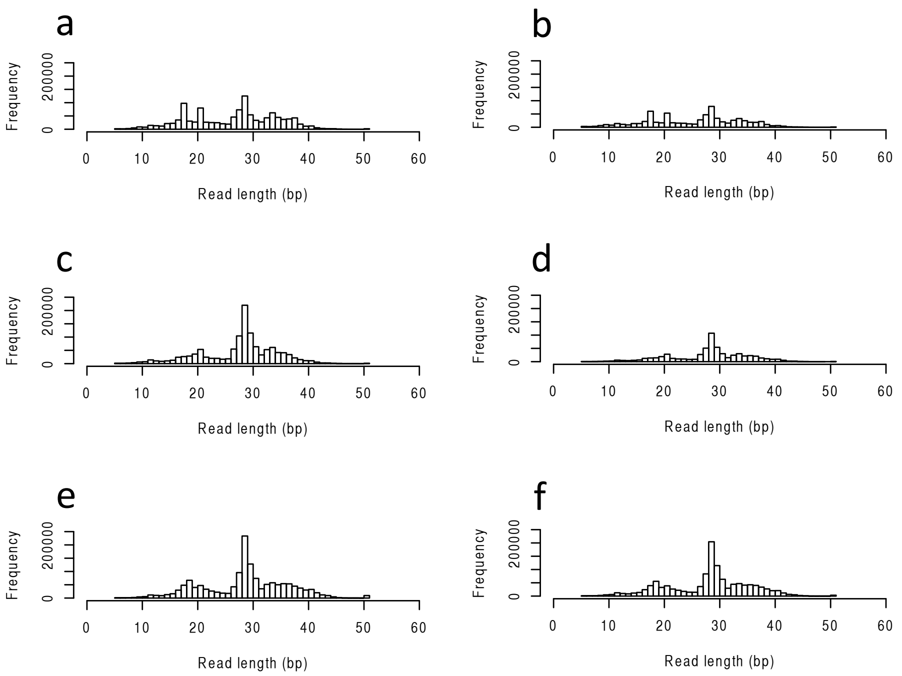

Figure S5: Histogram of length distribution of uniquely mapped sequenced reads from NGS for all the samples. a) S4A, b) S4B, c) S5A, d) S5B, e) S6A, f) S6B sample.

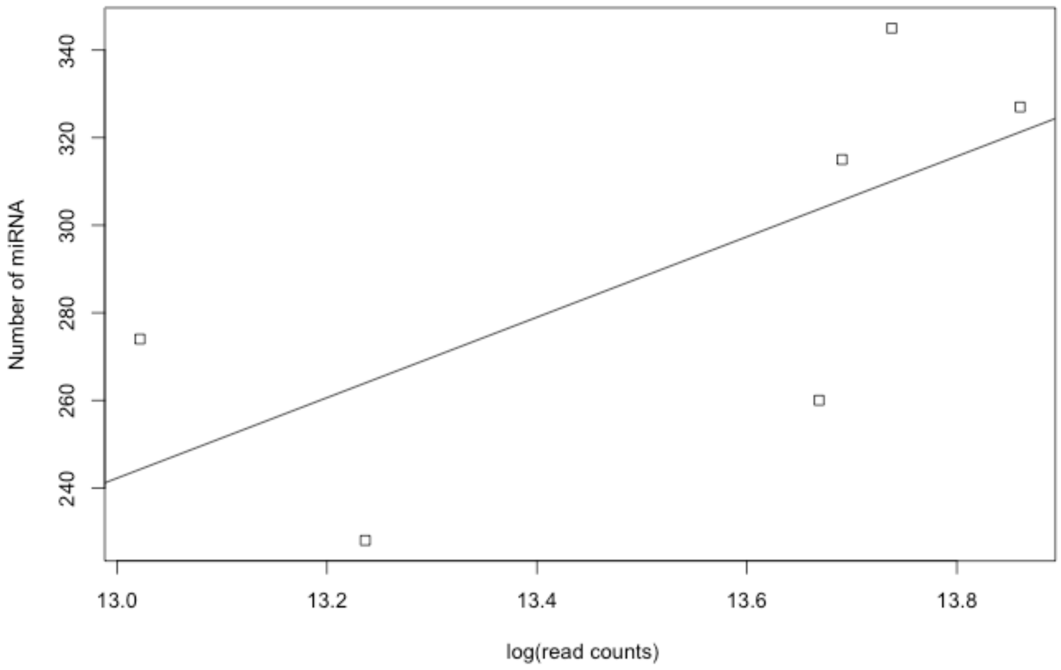

Figure S6: **Relationship of sequenced read counts per sample and detected miRNAs.** x-axis shows log of number of aligned reads per sample and y-axis shows the corresponding miRNA detected for that sample.

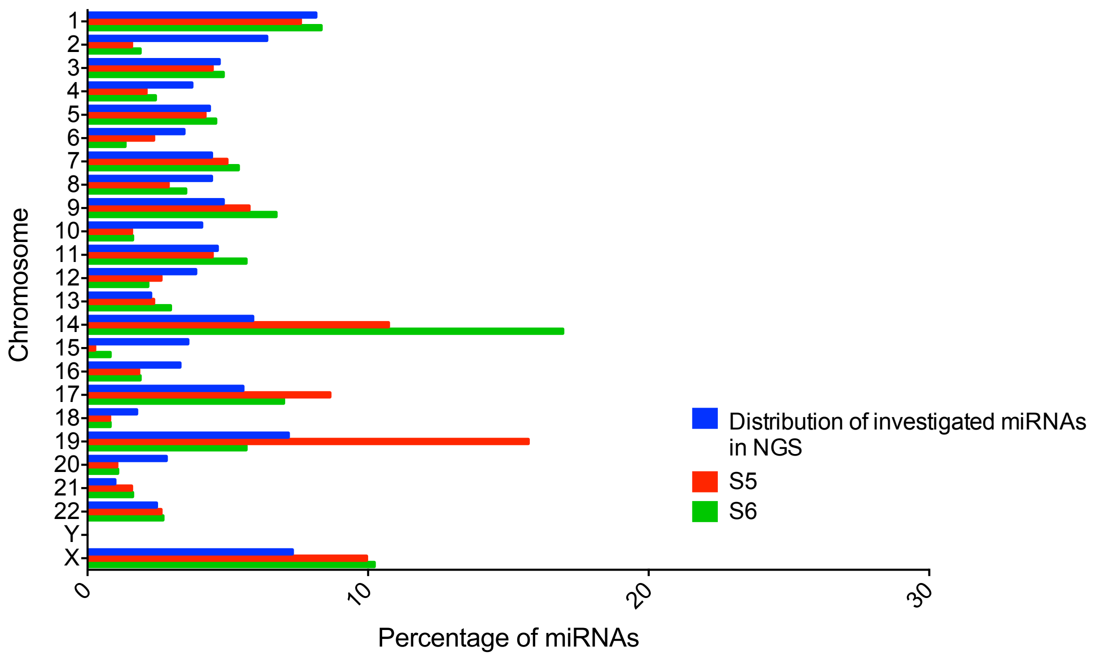

Figure S7: Comparative Chromosomal distribution of the detected miRNAs in the NGS platform. The 1735 known miRNAs from miRBase is shown in blue as the theoretical distribution.

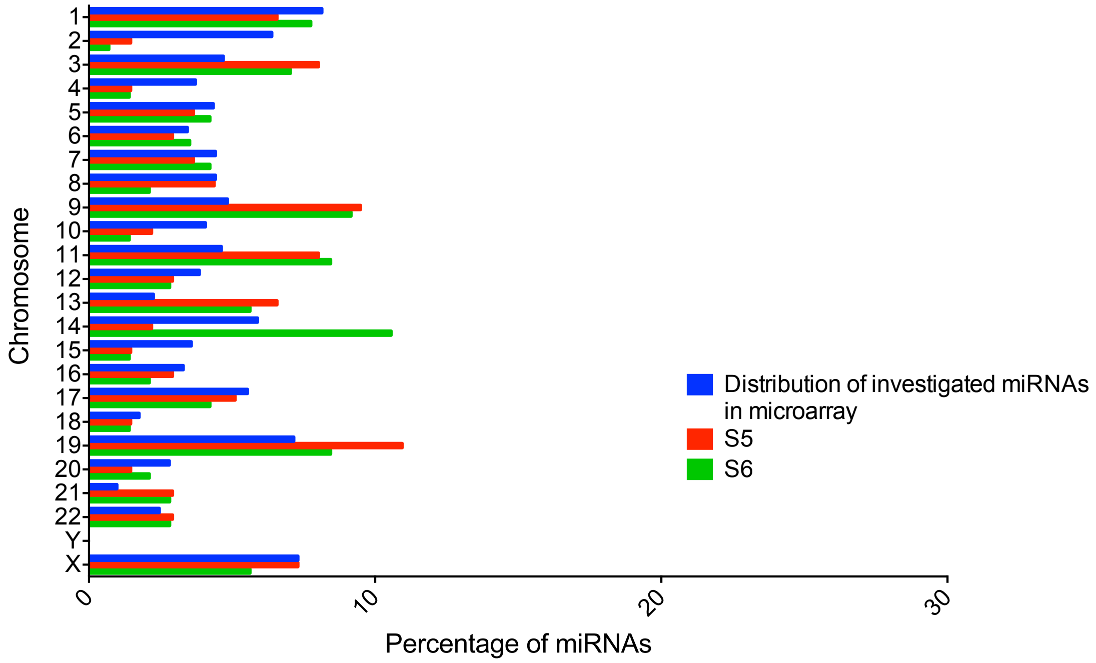

Figure S8: Comparative Chromosomal distribution of the detected miRNAs in the microarray platform. The 1735 miRNAs included in the microarray chip is shown in blue as the theoretical distribution.

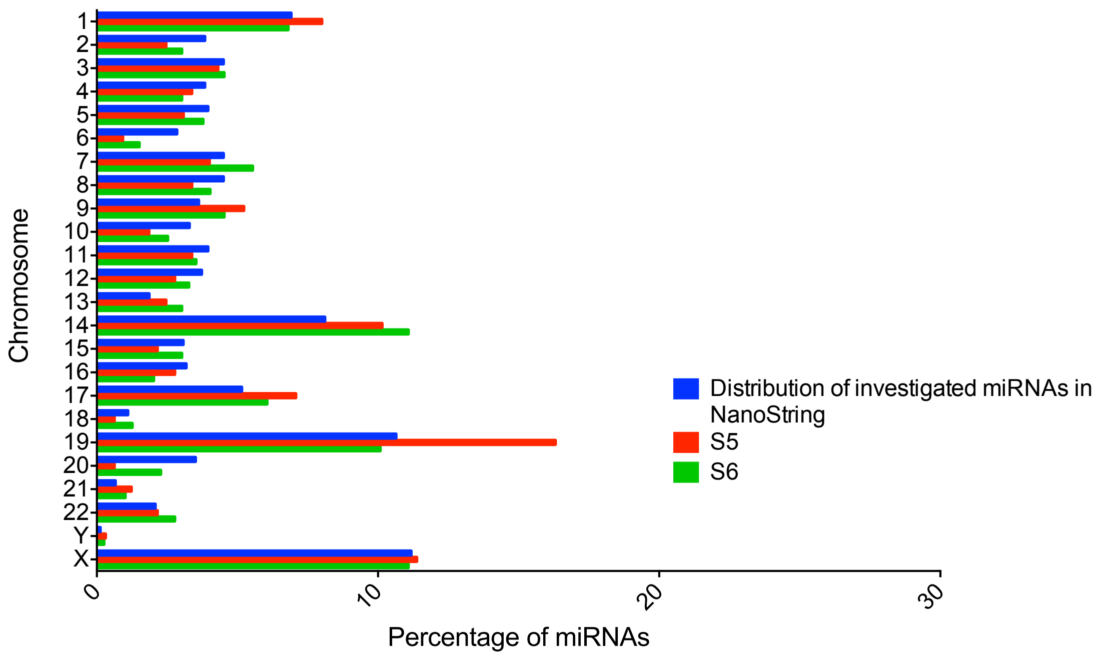

Figure S9: Comparative Chromosomal distribution of the detected miRNAs in the NanoString platform. The 800 known miRNAs included in the NanoString platform is shown in blue as the theoretical distribution.

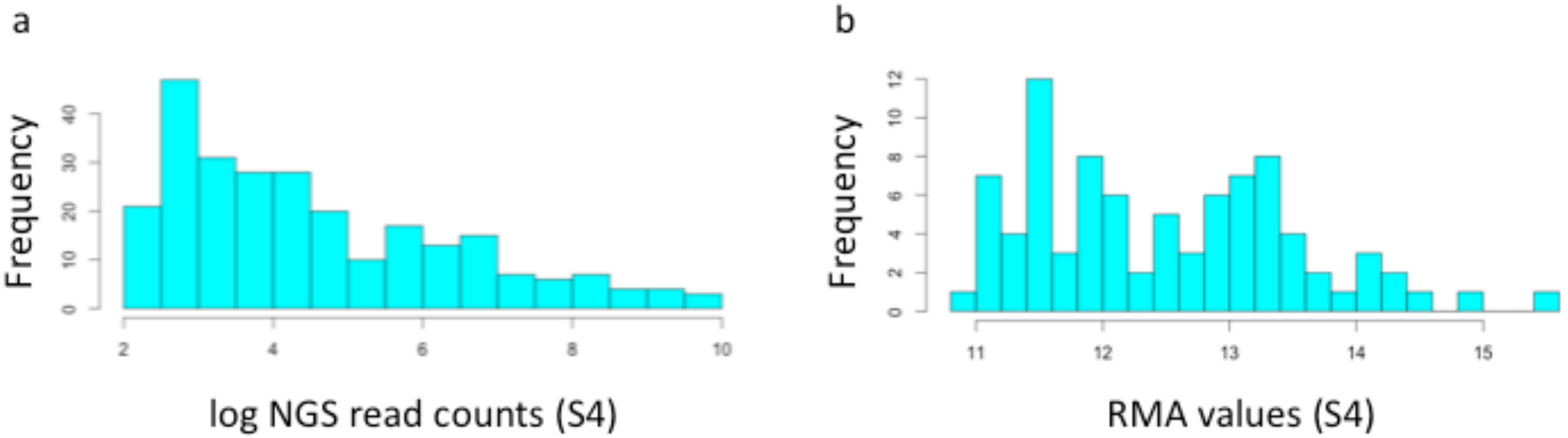

Figure S10: a) Distribution of the aligned sequenced read counts (log) and b) RMA values for the detected miRNA for S4 sample.

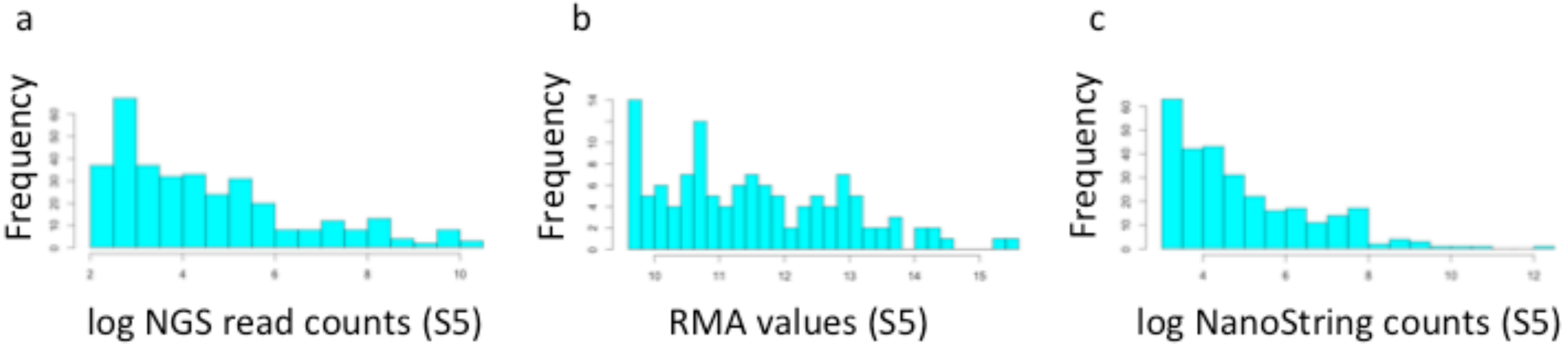

Figure S11: a) Distribution of the aligned sequenced read counts (log) and b) RMA values and c) Ncounter value for the detected miRNA for S5 sample.

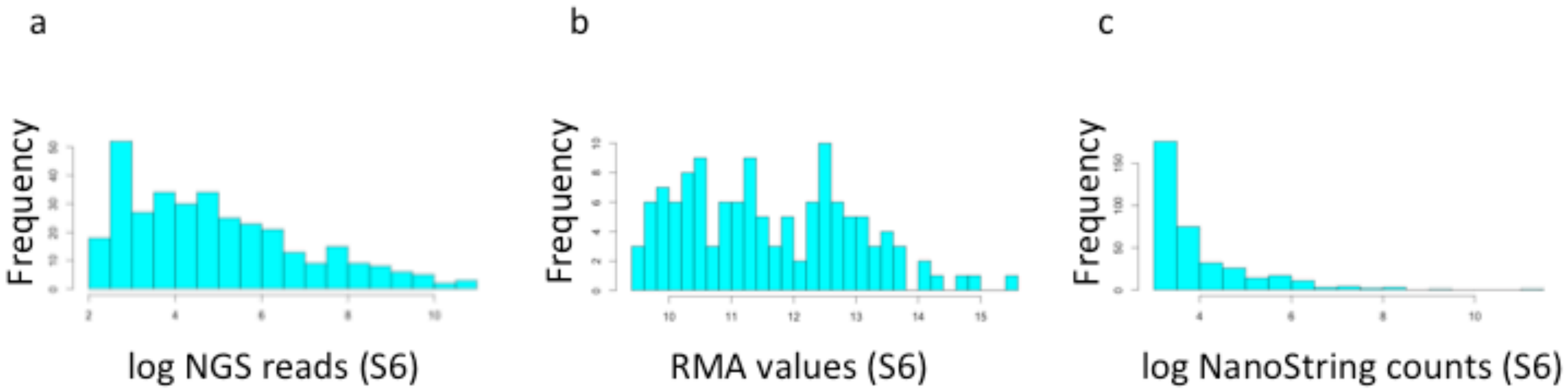

Figure S12: a) Distribution of the aligned sequenced read counts (log) and b) RMA values and c) Ncounter value for the detected miRNA for S6 sample.

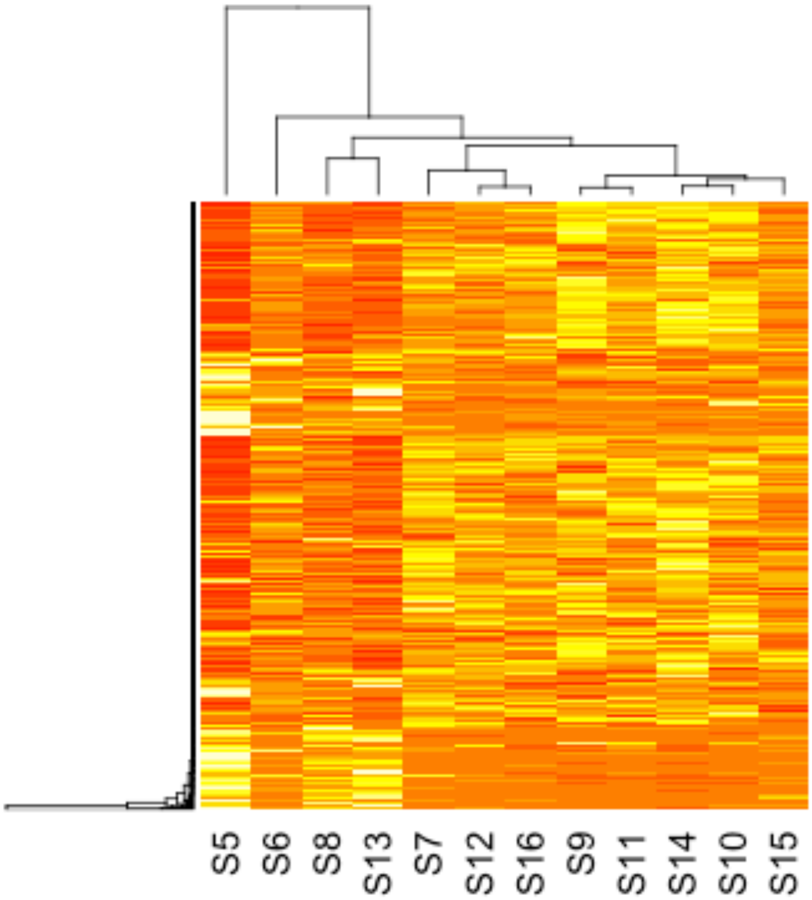

Figure S13: Hierarchical clustering of all the 800 miRNA for samples in Nanostring. A threshold for detection was not ap[illegible] producing this graph. (Function used in R: hclust (),method: complet[illegible] clustering, using a euclidean distance measurement). The heat ma[illegible] high (white/yellow) to low (red) expression of the miRNAs in different [illegible]

Additional Tables

Table S1: Clinical and histological details of the 10 additional samples used for profiling miRNA on the NanoString platform

Sample	Age	Tumour type	Tumour sub type	β- catenin mutation status	Comments on tumour	Location of phosphorylated β-catenin	Event free survival
S4	2 yrs	Epithelial/mesenchymal	Fetal	Absent	No teratoids	cytoplasm	3 yr 10 mnths
S5	5 mnths	Epithelial	Fetal	Absent	Cholangioblastic	cytoplasm	2 mnths
S6	8 mnths	Epithelial	Fetal	Present		cytoplasm	8 yr 11 mnths
S7	10 yrs 6 mnths	Epithelial	Fetal	Present	-	cytoplasm	1 yr 3 mnths
S8	1 yr 11 mnths	Epithelial	Fetal	Absent	Fibrosis post chemo	cytoplasm + nucleus	6 yrs
S9	2 yrs 3 mnths	Epithelial	Fetal	Absent	-	cytoplasm	8 yrs 4 mnths
S10	1 yr 6 mnths	Epithelial	Fetal/embryonal	Present	-	nucleus	7 yrs 6 mnths
S11	8 mnths	Epithelial	Fetal	Present	-	-	4 yrs 10 mnths
S12	1 yr 11 mnths	Epithelial/mesenchymal	Fetal/embryonal	Absent	-	cytoplasm	9 mnths
S13	2 yrs 4 mnths	Epithelial	Fetal	Absent	-	cytoplasm + nucleus	
S14	6 mnths	Epithelial	Fetal/embryonal	Absent	-	cytoplasm	5 yrs 3 mnths
S15	2 yrs 1 mnth	Epithelial	Fetal	Absent	-	cytoplasm	4 yrs 6 mnths
S16	4 mnths	Epithelial	Fetal	Absent	-	cytoplasm + nucleus	11 yrs

Table S2. Individual sample read counts before and after adapter removal and alignment efficiency

Sample	Number of reads (prior to adapter removal)	Number of reads (after adapter removal)	Unique alignment efficiency (%)
S4A	1.2 million	8.3 hundred thousand	31.0
S4B	7.5 hundred thousand	5.5 hundred thousand	29.0
S5A	1.3 million	9.1 hundred thousand	60.0
5SB	6.4 hundred thousand	4.4 hundred thousand	59.0
S6A	1.5 million	1.0 million	55.0
S6B	1.3 million	8.6 hundred thousand	56.0

Table S3. Correlation between technical replicates in the next generation sequencing platform.

Sample	Pearson correlation (read threshold ≥ 5)	Pearson correlation (read threshold ≥ 10)
S4A vs S4B	0.990	0.992
S5A vs S5B	0.992	0.995
S6A vs S6B	0.994	0.995

Table S4. Correlation between technical replicates in the microarray platform.

Sample	Pearson correlation (without filtering of RMA values)	Pearson correlation (after filtering of RMA values)$^{\#}$
S4A vs S4B	0.985	0.992
S5A vs S5B	0.992	0.998
S6A vs S6B	0.988	0.980

$^{\#}$ RMA filtering was performed using negative controls as a threshold for miRNA identification.

Table S5. Shared detection level of miRNAs in the three platforms

Platforms	S4		S5		S6	
	all	top 25%	all	top 25%	all	top 25%
NGS vs. MA	58	41	73	71	68	59
NGS vs. NS	-	-	71	69	58	60
MA vs. NS	-	-	76	74	69	66

NGS= next generation sequencing platform, MA= microarray, NS=NanoString. While calculating the overlap %, the platform with lower number of miRNA was taken as the denominator.

Table S6. Significance of shared miRNA detection level between platforms.

Platforms	S4 (P-value)		S5 (P-value)		S6 (P-value)	
	all	top 25%	all	top 25%	all	top 25%
NGS vs. MA	3.73e-21	3.23e-8	6.43e-40	2.29e-23	2.64e-36	2.71e-18
NGS vs. NS	-	-	1.79e-34	4.83e-40	8.26e-9	3.17e-31
MA vs. NS	-	-	1.97e-21	3.23e-19	2.63e-8	1.24e-13

Table S7. Concordance of detection for NanoString with NGS and MA after exclusion of absent probes[#]

Platform comparison	S5 (%)	S6 (%)
NGS vs. NS	76	72
MA vs. NS	89	86

For this comparison the miRNA and MA detected miRNA that were not included in the NanoString chip was excluded and then overlap % was recalculated.

Table S8. List and chromosome location of miRNAs detected by all three platforms

	Chromosome	miRNA		Chromsome	miRNA
1.	X	hsa-miR-532-5p[#]	42.	13	hsa-miR-20a
2.	X	hsa-miR-361-5p	43.	13	hsa-miR-18a
3.	X	hsa-miR-222	44.	13	hsa-miR-17
4.	X	hsa-miR-221	45.	13	hsa-miR-15a[#]
5.	X	hsa-miR-20b	46.	12	hsa-let-7i
6.	X	hsa-miR-10a[#]	47.	11	hsa-miR-483-5p
7.	X	hsa-miR-106a	48.	11	hsa-miR-192
8.	22	hsa-miR-185	49.	11	hsa-miR-130a
9.	22	hsa-let-7b	50.	11	hsa-miR-100
10.	21	hsa-miR-99a	51.	10	hsa-miR-146b-5p[#]
11.	21	hsa-miR-155	52.	10	hsa-miR-107
12.	21	hsa-let-7c	53.	9	hsa-miR-455-3p
13.	19	hsa-miR-99b	54.	9	hsa-miR-27b
14.	19	hsa-miR-517a[#]	55.	9	hsa-miR-23b
15.	19	hsa-miR-512-3p	56.	9	hsa-miR-199b-3p
16.	19	hsa-miR-27a	57.	9	hsa-miR-126
17.	19	hsa-miR-23a	58.	9	hsa-let-7d
18.	19	hsa-miR-150[#]	59.	8	hsa-miR-320a
19.	19	hsa-miR-125a-5p	60.	8	hsa-miR-30d[#]
20.	19	hsa-let-7e	61.	8	hsa-miR-30b
21.	18	hsa-miR-122	62.	8	hsa-miR-151-5p
22.	17	hsa-miR-497[#]	63.	8	hsa-miR-151-3p[#]
23.	17	hsa-miR-423-5p[#]	64.	7	hsa-miR-93
24.	17	hsa-miR-22	65.	7	hsa-miR-29a
25.	17	hsa-miR-21[#]	66.	7	hsa-miR-25
26.	17	hsa-miR-195	67.	7	hsa-miR-106b
27.	17	hsa-miR-152	68.	6	hsa-miR-30a
28.	16	hsa-miR-193b[#]	69.	5	hsa-miR-378[+]
29.	16	hsa-miR-140-3p	70.	5	hsa-miR-146a
30.	14	hsa-miR-494[+]	71.	5	hsa-miR-145
31.	14	hsa-miR-487b	72.	5	hsa-miR-143
32.	14	hsa-miR-487a[+]	73.	4	hsa-miR-574-3p
33.	14	hsa-miR-432[+]	74.	4	hsa-miR-4454
34.	14	hsa-miR-409-3p[+]	75.	3	hsa-miR-425
35.	14	hsa-miR-382	76.	3	hsa-miR-28-5p
36.	14	hsa-miR-379[+]	77.	3	hsa-miR-28-3p[#]
37.	14	hsa-miR-376c[+]	78.	3	hsa-miR-191
38.	14	hsa-miR-376a[+]	79.	3	hsa-miR-15b
39.	14	hsa-miR-342-3p	80.	3	hsa-let-7g
40.	14	hsa-miR-324-5p	81.	1	hsa-miR-34a
41.	14	hsa-miR-127-3p	82.	1	hsa-miR-320b[+]

	Chromosome	miRNA
83.	1	hsa-miR-214
84.	1	hsa-miR-205[+]
85.	-	hsa-miR-92a*
86.	-	hsa-miR-30c*[#]
87.	-	hsa-miR-26a*
88.	-	hsa-miR-24*
89.	-	hsa-miR-19b*
90.	-	hsa-miR-199a-5p*
91.	-	hsa-miR-199a-3p*
92.	-	hsa-miR-194*
93.	-	hsa-miR-181b*
94.	-	hsa-miR-181a*
95.	-	hsa-miR-16*
96.	-	hsa-miR-125b*
97.	-	hsa-let-7f*
98.	-	hsa-let-7a*

*indicates there is more than one stem loop which can produce this mature miRNA transcript; these stem loops are located on different chromosomes and therefore a single chromosome can not be assigned to the miRNA. [#]indicates this miRNA was found in all three platforms in S5 only. [+]indicated this miRNA was found in all three platforms in S6 only.

Table S9. Pairwise comparisons of S5 and S6 with additional 10 samples for percentage of overlap between commonly detected miRNAs

Sample vs. sample	Commonly detected miRNAs (%)	P-value (hypergeometric test)
S5/S6	78	9.99e-45
S5/S7	66	3.40e-24
S5/S8	99	2.03e-66
S5/S9	60	9.93e-19
S5/S10	60	2.18e-20
S5/S11	84	3.52e-35
S5/S12	69	1.34e-27
S5/S13	98	3.09e-96
S5/S14	62	5.82e-6
S5/S15	88	6.61e-51
S5/S16	65	2.77e-30
S6/S7	90	4.17e-56
S6/S8	96	7.66e-43
S6/S9	79	3.26e-55
S6/S10	82	6.72e-64
S6/S11	95	1.17e-39
S6/S12	79	3.61e-63
S6/S13	89	7.64e-45
S6/S14	77	5.65e-42
S6/S15	96	2.32e-53
S6/S16	88	7.47e-65
